# Role of the Genes of Type VI Secretion System in Virulence of Rice Bacterial Brown Stripe Pathogen *Acidovorax avenae* subsp. *avenae* Strain RS-2

**DOI:** 10.3390/ijms18102024

**Published:** 2017-09-21

**Authors:** Md. Mahidul Islam Masum, Yingzi Yang, Bin Li, Ogunyemi Solabomi Olaitan, Jie Chen, Yang Zhang, Yushi Fang, Wen Qiu, Yanli Wang, Guochang Sun

**Affiliations:** 1 State Key Laboratory of Rice Biology, Ministry of Agriculture Key Lab of Molecular Biology of Crop Pathogens and Insects, Institute of Biotechnology, Zhejiang University, Hangzhou 310058, China; masum@bsmrau.edu.bd (M.M.I.M.); 21416093@zju.edu.cn (Y.Y.); libin0571@zju.edu.cn (B.L.); solabomiolaitan@gmail.com (O.S.O.); 21616122@zju.edu.cn (J.C.); 11416065@zju.edu.cn (Y.Z.); 11416064@zju.edu.cn (Y.F.); 2 Department of Plant Pathology, Bangabandhu Sheikh Mujibur Rahman Agricultural University, Gazipur 1706, Bangladesh; 3 State Key Laboratory Breeding Base for Zhejiang Sustainable Pest and Disease Control, Institute of Plant Protection and Microbiology, Zhejiang Academy of Agricultural Sciences, Hangzhou 310021, China; wylcnrri@gmail.com

**Keywords:** *Acidovorax avenae* subsp. *Avenae*, T6SS, gene knock-out, pathogenicity, growth, biofilm, motility, Hcp

## Abstract

The Type VI secretion system (T6SS) is a class of macromolecular machine that is required for the virulence of gram-negative bacteria. However, it is still not clear what the role of T6SS in the virulence of rice bacterial brown stripe pathogen *Acidovorax avenae* subsp. *avenae* (Aaa) is. The aim of the current study was to investigate the contribution of T6SS in Aaa strain RS2 virulence using insertional deletion mutation and complementation approaches. This strain produced weak virulence but contains a complete T6SS gene cluster based on a genome-wide analysis. Here we compared the virulence-related phenotypes between the wild-type (RS-2) and 25 T6SS mutants, which were constructed using homologous recombination methods. The mutation of 15 T6SS genes significantly reduced bacterial virulence and the secretion of Hcp protein. Additionally, the complemented 7 mutations Δ*pppA*, Δ*clpB*, Δ*hcp*, Δ*dotU*, Δ*icmF*, Δ*impJ*, and Δ*impM* caused similar virulence characteristics as RS-2. Moreover, the mutant Δ*pppA*, Δ*clpB*, Δ*icmF*, Δ*impJ* and Δ*impM* genes caused by a 38.3~56.4% reduction in biofilm formation while the mutants Δ*pppA*, Δ*clpB*, Δ*icmF* and Δ*hcp* resulted in a 37.5~44.6% reduction in motility. All together, these results demonstrate that T6SS play vital roles in the virulence of strain RS-2, which may be partially attributed to the reductions in Hcp secretion, biofilm formation and motility. However, differences in virulence between strain RS-1 and RS-2 suggest that other factors may also be involved in the virulence of Aaa.

## 1. Introduction

The gram-negative bacteria *Acidovorax avenae* subsp. *avenae* (Aaa) causes diseases in a wide range of economically important plants such as rice, corn, oats, sugarcane, millet, and foxtail [1]. Particularly, this well-known seed-borne bacterium [1,2] causes bacterial brown stripe (BBS) in rice, which leads to heavy economic losses. It has been reported in many rice-growing countries such as Asia, Africa, North America and Europe [3,4]. The contaminated seeds represent the most important primary source of inocula [2,5,6] for outbreak of the disease. Recently, this disease has achieved increased attention, especially in China [7,8,9,10]. The economic importance of BBS makes it necessary to know the molecular basis for the infection of *A. avenae* subsp. *avenae* [9,11] to rice plants.

**Figure 1:**
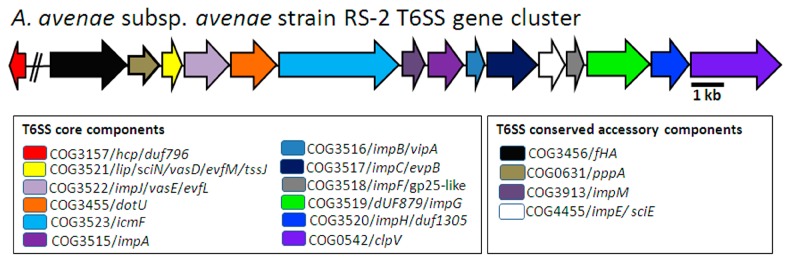
Schematic diagram of the genetic organization of *Acidovoraxavenae* subsp. *avenae* strain RS-2 T6SS gene cluster. Genes are indicated by arrows and the direction of the arrows represent the direction of transcription of the genes in genome. “//” indicates the presence of other genes but not belongs to T6SS. The database of Clusters of Orthologous Groups of proteins (COGs) was achieved from the National Center of Biotechnology Information (ftp://ftp.ncbi.nih.gov/pub/COG/COG2014/static/lists/listAciave.html).

The bacterial phytopathogens infections are highly associated with secreted effectors proteins in their host plants. It has been well documented that bacteria use a remarkable array of sophisticated macromolecular nanomachines to deliver extracellular proteins or effectors molecules into the surrounding environment or, in some cases, directly to the target site of the host cell [12,13]. At least six different secretion systems have been found in gram-negative pathogenic bacteria [12,14]. In particular, a novel secretary system T6SS has been described to be largely association with various biological functions such as pathogenicity, biofilm formation, adaptation, modulation of quorum sensing and survival [15,16,17,18]. Furthermore, according to our previous studies that the plant height was closely associated with bacterial virulence while T6SS genes showed differential responses to in vivo infection of Aaa to rice host [11,19]. Interestingly, genome-wide in silico analysis has identified a large number of secretion system-related genes including T6SS (Figure 1) in Aaa strain RS-2. However, the role of each gene of the T6SS gene cluster in Aaa strain RS-2 remains poorly understood.

The aim of this study was to examine the role of T6SS in Aaa strain RS-2 through comparing bacterial pathogenicity to rice, growth measurement, swimming motility, biofilm formation, and the secretion of effector proteins between wild-type and the mutants constructed in this study.

## 2. Results

### 2.1. In Silico Identification of T6SS Genes

Recently, the whole genome of *A. avenae* subsp. *avenae* strain RS-1 [7] and RS-2 (unpublished)-isolated from rice-has been sequenced by our laboratory, which is a useful resource for identifying genes involved in some specific biological functions underlying this disease. In this study, we found 25 Type VI secretion system genes in the *A. avenae* subsp. *avenae* strain RS-2 genome while the identification of T6SS homologous coding loci were conducted by local BLAST (BLASTN, BLASTX) and results are shown in Supplementary Figure S1. Based on the genome wide analyses of *A. avenae* subsp. *avenae* strain RS-1, *Acidovorax avenae* subsp. *avenae* ATCC 19860 and *A. citrulli* AAC00-1, one T6SS gene cluster was also found in Aaa strain RS-2 (Figure 1).

As shown in Figure 1, this cluster contains 16 genes namely *hcp*, *fHA*, *pppA*, *lip*, *impJ*, *dotU*, *icmF*, *impM*, *impA*, *impB*, *impC*, *impE*, *impF*, *dUF879*, *impH*, and *clpV*, which have been shown to represent core and conserved accessory components in the T6SS of Aaa strain RS-2. Phylogenetic analysis of sequences in this study clearly indicated the presence of T6SS genes in *A. avenae* subsp. *avenae* strain RS-2, which were homologs in closely related bacteria such as *A. avenae* subsp. *avenae* strain RS-1 and ATCC 19860 as well as *A. citrulli* AAC00-1 (Supplementary Figure S2). All T6SS genes in the Aaa strain RS-2 genome including their putative functions are presented in the Supplementary Table S1.

### 2.2. T6SS Gene Mutants and Complementation

**Figure 2:**
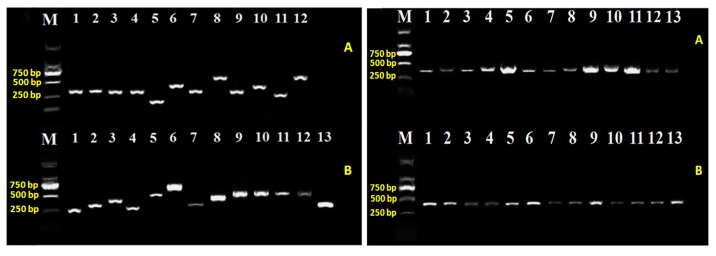
Validation of the 25 T6SS using specific primers of each gene (left) and specific primer of *Acidovorax avenae* subsp. *avenae* strain RS-2 mutants (right); M: Marker DL2000; (**A**) 1~13: Δ*pppA*, Δ*clpB*, Δ*hcp*, Δ*fHA*, Δ*lip*, Δ*impJ*, Δ*dotU*, Δ*icmF*, Δ*impM*, Δ*impA*, Δ*impB*, Δ*impC*, the *w*ild-type; (**B**) 1~13: Δ*impE*, Δ*impF*, Δ*dUF879*, Δ*impH*, Δ*clpV*, Δ*vgrG-1*, Δ*vgrG-2*, Δ*vgrG-3*, Δ*vgrG-4*, Δ*vgrG-5*, Δ*vgrG-6*, Δ*vgrG-7*, Δ*vgrG-8.*

**Figure 3:**
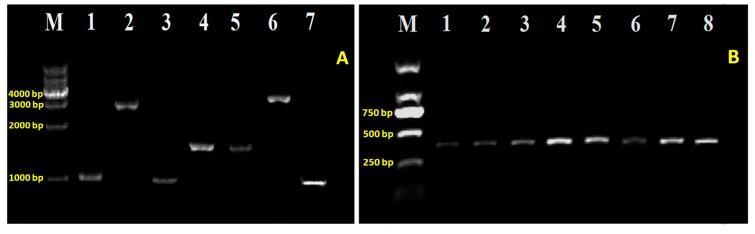
Validation for the complementation of T6SS mutants using specific primers of the 7 virulence-associated genes (left) and specific primer of *Acidovorax avenae* subsp. *avenae* strain RS-2 (right); (**A**) M: 1 kb DNA Ladder; 1~7: Δ*pppA*, Δ*icmF*, Δ*hcp*, Δ*dotU*, Δ*impJ*, Δ*clpB*, Δ*impM*; (**B**) M: Marker 2000; 1~7: Δ*pppA-*comp, Δ*icmF-*comp, Δ*hcp-*com*p*, Δ*dotU-*comp, Δ*impJ-*comp, Δ*clpB-*comp, Δ*impM-*comp, the wild-type.

To study the involvement of T6SS genes in virulence mechanism, we created the insertional knockout mutants on target genes of T6SS in Aaa strain RS-2 via homologous recombination (primers, constructed strains and plasmids were listed in Supplementary Tables S2 and S3). After successfully transforming the plasmid construct, transformants grown on selective antibiotic were ensured by PCR diagnosis for T6SS mutants (Figure 2) and their complementation as well (Figure 3). Subsequently, the identity of all the obtained strains were verified by blasting their sequence in the NCBI database, and the results showed the highest homology with Aaa (data not shown). The knockout mutants were then examined for phenotypes in several aspects such as pathogenicity, growth, swimming, biofilm formation, and secretion of effector proteins.

### 2.3. T6SS Affected Pathogenicity of Aaa Strain RS2 to Rice Seedlings

**Figure 4:**
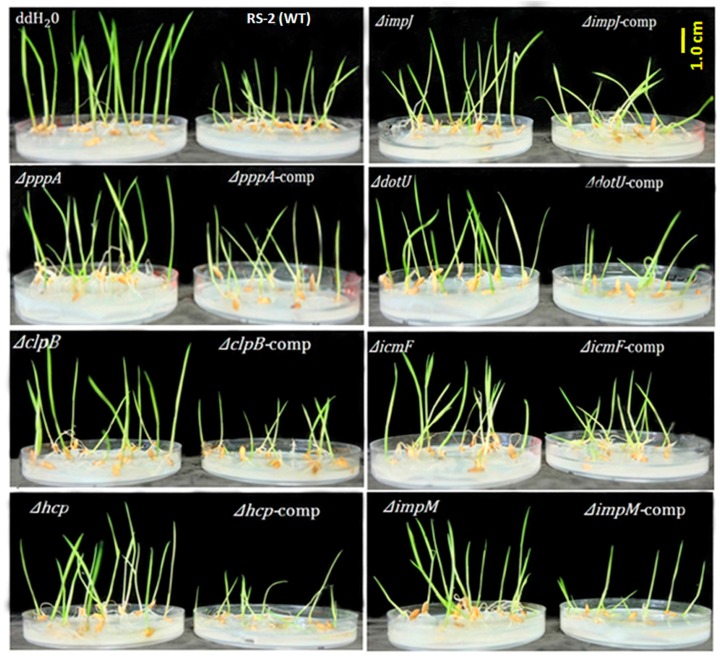
Seed-transmission assay for the virulence of the wild-type, the T6SS mutants and their corresponding complemented strains of *Acidovorax avenae* subsp. *avenae* strain RS-2 to rice seedling. Germinated rice seeds (cv. II You 023, *n* = 100/mutant) were inoculated with bacterial suspension of ~1×10^8^ colony forming units (CFU)/mL. Fifteen seeds were planted per sterile agar (0.5%) plate and seedlings were evaluated 5 days after planting on plate.

**Figure 5:**
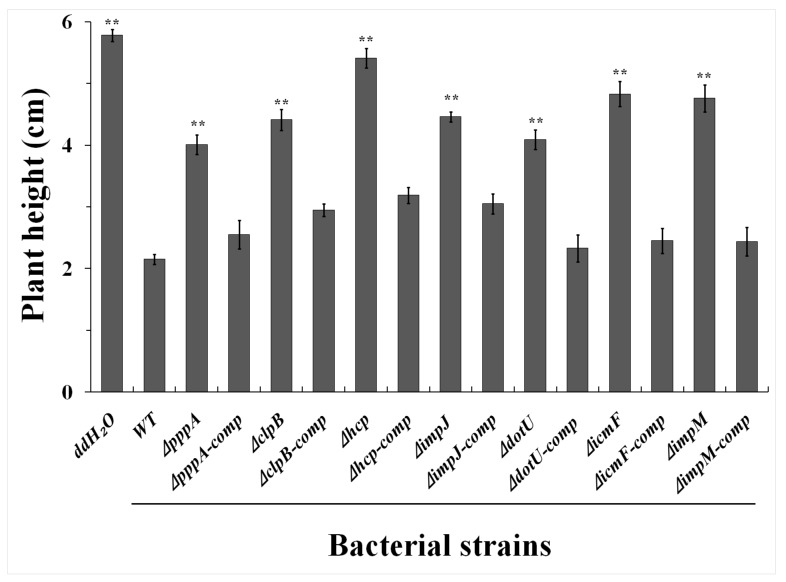
The effect of T6SS genes of *Acidovorax avenae* subsp. *avenae* strain RS-2 on virulence phenotype in respect of plant height for the rice seedling. WT: wild-type strain RS-2; ddH*_2_*O: double distilled H_2_O; Δ*pppA/*Δ*pppA*-comp: mutant/complementation of *pppA*; Δ*clpB/*Δ*clpB*-comp: mutant/complementation of *clpB*; Δ*hcp/*Δ*hcp*-comp: mutant/complementation of *hcp*; Δ*impJ/*Δ*impJ*-comp: mutant/complementation of *impJ*; Δ*dotU/*Δ*dotU*-comp: mutant/complementation of *dotU*; Δ*icmF/*Δ*icmF*-comp: mutant/complementation of *icmF*; Δ*impM/*Δ*impM*-comp: mutant/complementation of *impM*. Vertical bars represent standard errors of the means. Asterik symbol above the data bars indicate a significant difference between the wild-type and T6SS mutants, complemented strains or negative control (** *p* < 0.01).

The effect of T6SS genes on the virulence of the Aaa strain RS-2 was determined by comparing the plant height of rice seedlings between the wild-type and individual T6SS mutants, and results are presented in Figure 4 and Figure 5 and Supplementary Table S4.

As shown in Figure 4 and Figure 5, we observed that rice seedlings treated with wild-type strain RS-2 grows weaker and shorter with an average of only 2.15 cm whereas ddH_2_O (the negative control) treated seedlings showed the maximum plant height (5.78 cm). Furthermore, results showed that 7 of the mutants-Δ*pppA*, Δ*clpB*, Δ*hcp*, Δ*impJ*, Δ*dotU*, Δ*icmF* and Δ*impM*-caused the greatest (*p* < 0.01) reduction in bacterial virulence compared to Aaa wild-type RS-2 strain, while the corresponding average plant heights were 4.01, 4.41, 5.41, 4.46, 4.09, 4.83, 4.76 cm, respectively.

An association between plant height and bacterial virulence was revealed in the above result. As mentioned above, the wild-type strain RS-2 showed the strongest virulence toward rice seedlings, which reduced the plant height by 62.80% compared with that treated with the ddH_2_O, whereas the corresponding plant height in mutants Δ*pppA*, Δ*clpB*, Δ*hcp*, Δ*impJ*, Δ*dotU*, Δ*icmF* and Δ*impM* decreased by 30.62%, 23.70%, 6.40%, 22.84%, 29.24%, 16.44%, 17.65%, respectively (see Figure 4 and Supplementary Table S4).

Complementation of these mutants restored their virulence (Figure 4 and Figure 5). Besides the above mentioned 7 mutants, the other 8 mutants (Δ*vgrG* (1–8)) also caused a significant (*p* < 0.05) reduction in pathogenicity compared with the wild-type strain. Moreover, there was no significant (*p* > 0.05) difference in the pathogenicity between the 10 mutants (Δ*fHA*, Δ*clpV*, Δ*dUF879*, Δ*impA*, Δ*impB*, Δ*impC*, Δ*impE*, Δ*impF*, Δ*impH* and Δ*lip*) and the wild-type strain RS-2 (Supplementary Table S4).

In conclusion, among the 25 T6SS genes, the mutation of 15 of them had a significant effect on Aaa virulence in respect of rice plant height, and the other 10 genes did not. Based on this information, we can infer that the loss of pathogenicity of rice bacterial brown stripe disease in the mutants was mainly due to the deficiency of different T6SS genes.

### 2.4. T6SS Affected the Growth of Aaa Strain RS2

**Figure 6:**
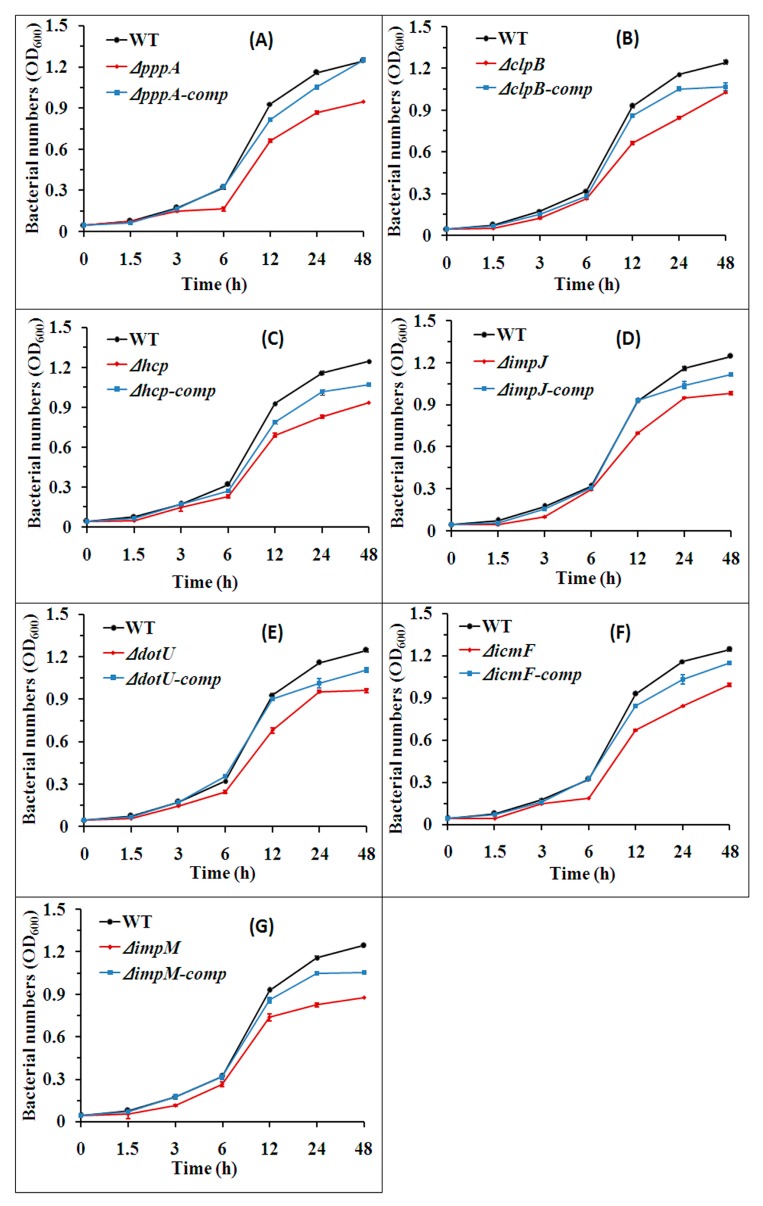
Bacterial growth of the wild-type, the T6SS mutants and their corresponding complemented strains of *Acidovorax avenae* subsp. *avenae* strain RS-2. (**A**–**G**) Indicate the comparisons between wild-type and the mutant/complementation of gene *pppA*, *clpB*, *hcp*, *impJ dotU*, *icmF* and *impM*, respectively. Means ± SEM are shown (*n* = 6). The experiment was repeated three times and here is presented one set of representative data.

Results from this study indicated that the strain RS-2 wild-type, its mutants and their complemented strains showed the fastest growth rate during incubation for 6~12 h, followed by moderate growth after incubation for 24 h, and then reached maximum growth rate after incubation for 48 h (Figure 6). Indeed, the OD_600_ of wild-type strain RS-2 was 0.076, 0.175, 0.322, 0.929, 1.158 and 1.245 after incubation for 1.5, 3.0, 6.0, 12.0, 24.0 and 48.0 h, respectively, while the mutation of seven T6SS genes Δ*pppA*, Δ*clpB*, Δ*hcp*, Δ*impJ*, Δ*dotU*, Δ*icmF*, Δ*impM* caused 29.03%, 48.38%, 25.81%, 24.73%, 26.88%, 27.96%, 20.43% reduction in bacterial growth after incubation for 12.0 h; 25.00%, 42.24%, 28.45%, 18.10%, 18.10%, 27.59% and 31.03% reduction in bacterial growth after incubation of 24 h; 24.00%, 16.00%, 24.80%, 21.60%, 23.20%, 20.80% and 29.6% reduction in bacterial growth after incubation for 48 h, respectively, compared to the wild-type strain RS-2 (Figure 6). However, there was no significant difference in the growth between the wild-type strain RS-2 and the complemented strains Δ*pppA*-comp, Δ*clpB*-comp, Δ*hcp*-comp, Δ*impJ*-comp, Δ*dotU*-comp, Δ*icmF*-comp, Δ*impM*-comp but slight reduction on the OD_600_ value for complements after culturing for 12 h (Figure 6). In addition, the OD_600_ values of the other 18 mutant strains were very close to that of the wild-type strain during the whole incubation time, indicating that there were no significant differences between these mutants and the wild-type (data not shown).

### 2.5. T6SS Affected the Biofilm Formation of Aaa Strain RS2

**Figure 7:**
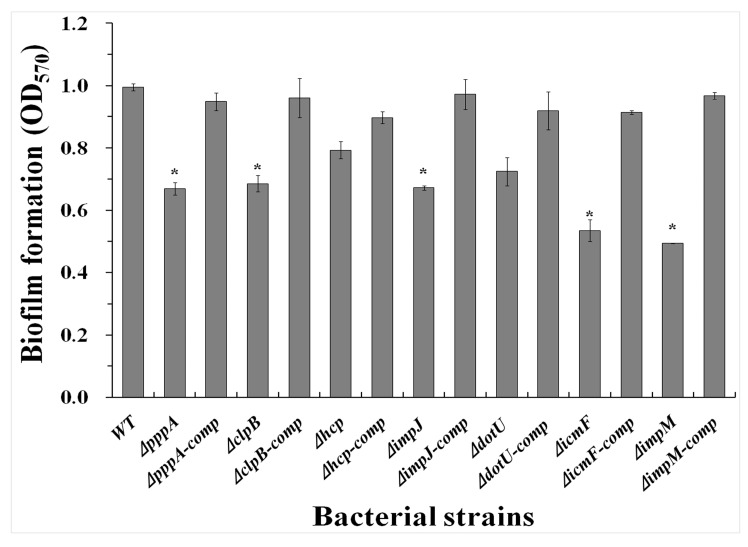
Quantification of biofilm produced by the wild-type, the T6SS mutants and the corresponding complemented strains of *Acidovorax avenae* subsp. *avenae* strain RS-2. Means ± SEM are shown (*n* = 6). Vertical bars represent standard errors of the means. Asterick indicated significant difference between the wild-type strain and the T6SS mutants, or the complemented strains (* 0.01 < *p* < 0.05).

After incubation at 30 °C for 48 h without agitation, the quantification of the biofilm confirmed that mutants Δ*pppA*, Δ*clpB*, Δ*impJ*, Δ*icmF*, and Δ*impM* reduced biofilm adhesion in microtitre plates while the optical density (OD_570_) value were measured and the results listed in Figure 7 were 0.67, 0.69, 0.79, 0.67, 0.72, 0.53 and 0.49, respectively. The wild-type, strain RS-2 produced a stronger biofilm while the OD_570_ value for stained biofilm was 0.99, which was significantly (*p* < 0.05) higher than that of Δ*pppA*, Δ*clpB*, Δ*impJ*, Δ*icmF*, and Δ*impM*.

However, mutants Δ*hcp* and Δ*dotU* had statistically similar OD_570_ value compared with RS-2 wild-type, indicating that *hcp* and *dotU* genes did not significantly affect the formation of biofilm. Similar to the wild-type strain, complementation of the mutants Δ*pppA*, Δ*clpB*, Δ*impJ*, Δ*icmF* and Δ*impM* showed compatible biofilm-forming capability (*p* > 0.05). Additionally, there were no significant (*p* > 0.05) differences in biofilm formation between the wild-type strain and the other single T6SS gene mutants of Aaa strain RS-2. Taken together, these results indicate that these T6SS genes might be involved in the biofilm formation of Aaa strainRS-2.

### 2.6. T6SS Affected the Swimming Motility of Aaa Strain RS2

**Figure 8:**
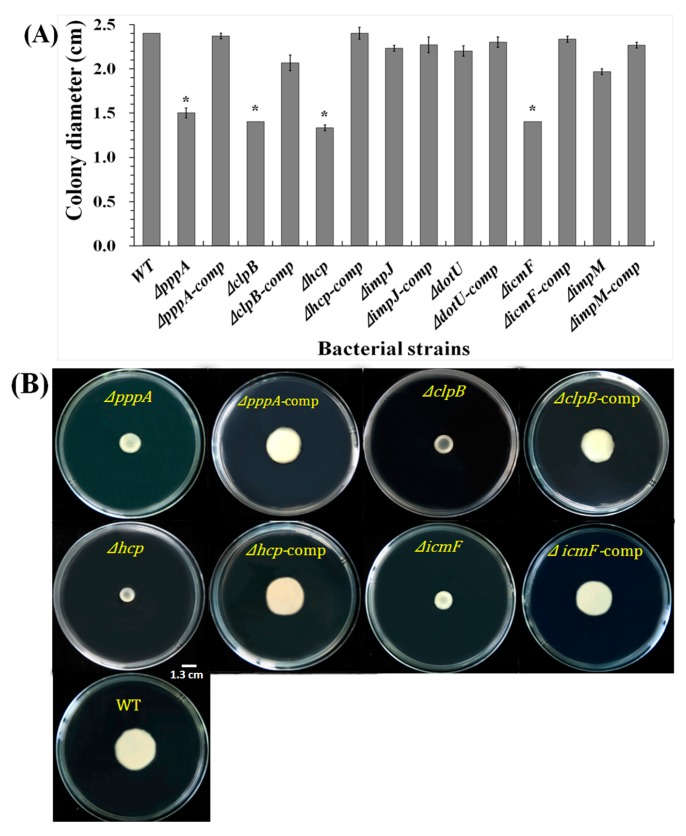
The mutation of the T6SS genes *pppA*, *clpB*, *hcp* and *icmF* inhibited the swimming motility of *Acidovorax avenae* subsp. *avenae* strain RS-2. Statistically significant difference (0.01 < * *p* < 0.05 by student’s *t*-test) was found between the T6SS mutants and the corresponding complemented strains or the wild-type (**A**). The swimming motility was determined by measuring the diameters of bacterial colony on the plates from three independent experiments (**B**).

The effects of T6SS on the motility of the Aaa strain RS2 were determined by measuring the diameter of the area covered by swimming bacteria on LB plates with 0.3% agar after 48 h of incubation and the results are shown in Figure 8.

Results from the swimming assays showed that mutant Δ*clpB*, Δ*hcp* and Δ*icmF* had lost motility that was restored upon complementation. The colony diameters of Δ*pppA*, Δ*clpB*, Δ*hcp* and Δ*icmF* were 1.5, 1.4, 1.33, and 1.4 cm, which were decreased by 37.5%, 41.66%, 44.59% and 41.66%, respectively compared with the wild-type strain RS-2, suggesting that these genes might be related to bacterial swimming. However, no significant difference was found between the colony diameter of the wild-type strain and the other mutants (Δ*impJ*, Δ*dotU* and Δ*impM)*, indicating that the three T6SS genes did not significantly affect the swimming of Aaa strain RS-2.

### 2.7. T6SS Affected Hcp Secretion of Aaa Strain RS2

**Figure 9:**
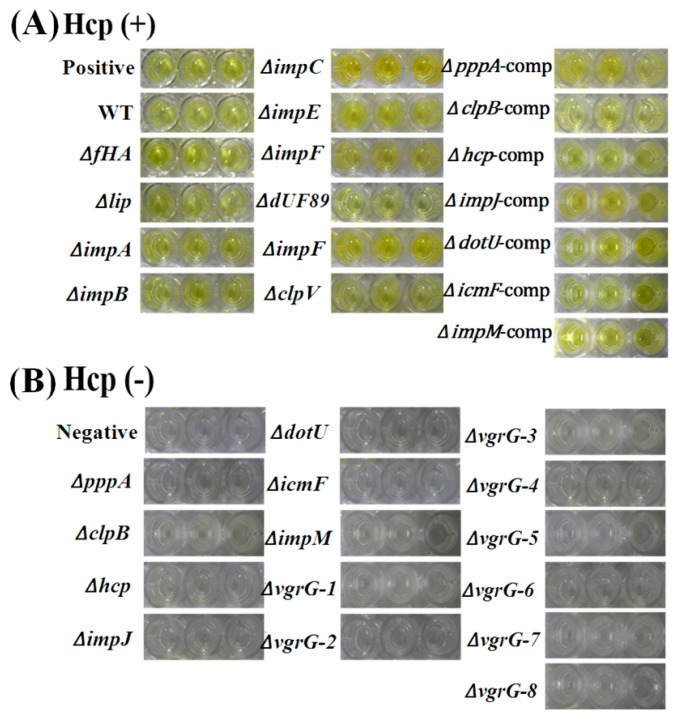
ELISA detection for the secretion of Hcp effector protein by the wild-type, the T6SS mutants and the corresponding complemented strains of *Acidovorax avenae* subsp. *avenae* strain RS-2.(**A**) The positive reaction (P/N value ≥ 2.1); (**B**) The negative reaction (P/N value < 2.1). The purified Hcp-His fusion protein and His protein were used as the positive and the negative control, respectively. The experiment was conducted three times with 3 replicates.

The effects of T6SS on Hcp effectors protein secretion were examined in the Aaa strain RS-2 based on an ELISA experiment with polyclonal rabbit serum, which was performed using the purified Hcp-His fusion protein at a dilution of 5000. Results are shown in Figure 9. It can be seen that there was a positive (P/N > 2.1) ELISA reaction for the mutants Δ*fHA*, Δ*lip*, Δ*impA*, Δ*impB*, Δ*impE*, Δ*impF*, Δ*dUF879*, Δ*impH*, Δ*clpV* and the wild-type*.* Furthermore, the optical density (OD_450_) for the Hcp secretion of Δ*fHA*, Δ*lip*, Δ*impA*, Δ*impB*, Δ*impE*, Δ*impF*, Δ*dUF879*, Δ*impH*, Δ*clpV* were 0.361, 0.368, 0.361, 0.359, 0.352, 0.350, 0.355, 0.351, 0.367, 0.364, respectively, while the OD_450_ of the wild-type strain was 0.413 (Figure 9). Obviously, these T6SS mutants did not reduce significantly the OD_450_ values (*p* ≤ 0.5) compared to the wild-type, indicating that mutations of these genes did not affect the secretion of Hcp protein in strain RS-2. In contrast, there was a negative reaction (P/N ≤ 1.5) for the other 15 mutants Δ*pppA*, Δ*clpB*, Δ*hcp*, Δ*impJ*, Δ*dotU*, Δ*icmF*, Δ*impM*, Δ*vgrG*-1, Δ*vgrG*-2, Δ*vgrG*-3, Δ*vgrG*-4, Δ*vgrG*-5, Δ*vgrG*-6, Δ*vgrG*-7 and Δ*vgrG*-8, while the OD_450_ values were significantly lower than those of the wild-type, indicating that the 15 genes may significantly affect the secretion of Hcp protein in the RS-2 strain. In addition, there was a positive ELISA reaction for the complementation Δ*pppA*-comp, Δ*clpB*-comp, Δ*hcp*-comp, Δ*impJ*-comp, Δ*dotU*-comp, Δ*icmF*-comp and Δ*impM*-comp, while they had similar OD_450_ values with the wild-type of the Aaa RS-2 strain.

## 3. Discussion

T6SSs have been shown to be involved in the virulence of a wide range of gram-negative bacteria including plant and animal pathogens such as *Edwardsiella tarda*, *Vibrio cholera*, *Pseudomonas aeruginosa*, *Burkholderia mallei*, avian pathogenic *Escherichia coli*, *Xanthomonas oryzae*, *Pectobacterium atrosepticum* and *Agrobacterium tumefaciens* [14,20,21,22,23,24,25]. The T6SS is typically encoded by clusters of contiguous genes and composed of at least 13 conserved proteins along with a variable complement of accessory elements [21,26]. Gram-negative bacteria utilize T6SS as a functional apparatus to expel virulence factors from cytoplasm to the extracellular surroundings and within a eukaryotic target cell for their virulence and survival in hosts [14,20,21,27,28].

In the current study, 25 T6SS genes that are highly homologous to that of the Aaa strain RS-1 were identified in the Aaa strain RS-2 based on genome-wide in silico analysis. Furthermore, the involvement of these T6SS genes in the pathogenicity of Aaa strain RS-2 was justified by the difference in virulence-related phenotypes between the constructed mutants and the wild-type. In agreement with the results of this study, our previous studies not only confirmed the in silico prediction of T6SS component OmpA/MotB and ClpB based on LC-MS/MS analysis of outer membrane proteins in theAaa strain RS-1, but also revealed that T6SS have a strong response to in vivo infection and different stresses based on RNA-Seq analysis [26,29]. These results highlighted that T6SS are highly associated with the virulence of rice bacterial brown stripe pathogen Aaa.

In general, this study found that there were significant differences in virulence among the 25 T6SS genes mutants of the Aaa strain RS-2. This is consistent with the results of our previous studies, which indicated that T6SS genes had differential responses to in vivo infection of the Aaa strain RS-1 to rice host [8,11,29,30]. Furthermore, this study indicated that the virulence of the Aaa strain RS-2 was significantly reduced by the mutation of 15 T6SS genes, but unaffected by the other T6SS genes based on seed transmission assays. This reveals the complexity of the T6SS genes in the virulence of the Aaa strain RS-2, which makes it necessary to examine the function of each T6SS gene in these kind of studies. Indeed, T6SS has been also reported to be involved in a series of cellular activities such as translocation function, stress resistance, extracellular protease production and intracellular communication, which are unrelated to pathogenicity [16,20,21,23]. However, this study showed that 15 T6SS genes of strain RS-2 are highly associated with the virulence of Aaa.

In agreement with the results of this study, Sheng et al. [31] reported that the *pppA* contributes to virulence actions such as biofilm formation, motility, and cell aggregation. Zhang et al. [19] demonstrated that the deletion of *clpB* significantly affected bacterial growth, virulence, exopolysaccharide production, biofilm formation of the Aaa strain RS-1. Furthermore, the IcmF-DotU complex consists of the main transmembrane structure in the needle-like T6SS apparatus [15,32]. Thus, the contribution of *dotU* and *icmF* to bacterial virulence was mainly due to their regulation in secretion of the putative effector proteins haemolysin co-regulated proteins (Hcp) and valine-glycine repeat (VgrG), which have been shown to play an important role in bacterial virulence [12,14]. In addition, the specific functions of *impJ* and *impM* in T6SS is still not known, although *impJ* has been regarded as a trimeric cytoplasmic protein that interacts with the membrane and phage-like complexes in T6SS [33] and *impM* is responsible for encoding the member of a Type VI secretion-associated protein that belongs to theBMA_A0400 family in T6SS containing bacteria [14].

Furthermore, results of the ELISA experiment indicated that no Hcp effector protein was detected in the secretory proteins from the mutants of 15 virulence -associated T6SS genes, while their complementation showed a positive reaction similar to the wild-type strain RS-2. This result is consistent with previous studies, which found that T6SS has also been reported to function through the secretion of effector proteins such as Hcp. Indeed, besides its structural function in T6SS, the secretion of Hcp is thought to be an effector protein as the hallmark of a functional T6SS in many bacteria including *V. cholerae*, *A. hydrophila*, *P. aeruginosa* and *B. pseudomallei*, *E. coli*, *A. tumefaciens* [12,27,34,35,36]. Therefore, the results of this study supported the evidence that the role of T6SS in bacterial virulence is highly associated with the secretion of effector Hcpin strain RS-2.

However, there was a difference in pathogenicity among the 15 virulence-associated T6SS genes, while the mutation of the 7 T6SS genes Δ*pppA*, Δ*clpB*, Δ*hcp*, Δ*impJ*, Δ*dotU*, Δ*icmF*, Δ*impM* caused a greater reduction in plant height than that of the 8 Δ*vgrG* (1–8) genes, revealing that the other virulence determinants such as growth, biofilm, swimming other than Hcp effector proteins may also be attributed to the function of T6SS. Indeed, the greater reduction in virulence may be at least partially explained by the result that bacterial growth was impaired by the 7 T6SS genes mutants, but was unaffected by the 8 Δ*vgrG* (1–8) genes mutants compared with the wild-type. Interestingly, mutation of the 7 T6SS genes showed a differential effect on bacterial swimming and biofilm formation, which has been widely reported to be highly associated with bacterial virulence [11,16,37,38,39]. Furthermore, in agreement with the results of this study, the T6SS gene clusters have been shown to be involved in the biofilm formation of other bacteria such *A. citrulli* [6], *P. fluorescens* [40], *V. alginolyticus* [16] and *V. parahaemolyticus* [41]. This result reveals the complexity of T6SS in the virulence mechanism of the Aaa strain RS-2.

## 4. Materials and Methods

### 4.1. Bacterial Strains, Growth Media and Inoculum Preparation

The bacterial strains and plasmids used in this study are described in Supplementary Table S2. The Aaa wild-type strain RS-2 and mutant strains were cultured in Luria–Bertani (LB) agar or broth medium [42] at 30 °C. *Escherichia coli* strains were grown in LB agar or broth medium at 37 °C. For inoculation, bacterial strains were grown in LB broth for 48 h, serially diluted into ddH_2_O, and the final concentration of bacterial suspension was adjusted to an approximate optical density (OD_600_) of 0.6 (~1 × 10^8^ CFU/mL) with a spectrophotometer (Perkin Elmer Lambda 35 UV/VIS, Waltham, MA, USA). When required, the culture media were supplemented with the following concentrations: ampicillin (Amp), 100 µg mL^−1^; kanamycin (Km), 50 µg mL^−1^; rifampicin (Rif), 50 µg mL^−1^; and Chloramphenicol (Chl), 3.4 µg mL^−1^.

### 4.2. DNA Extraction and Amplification

Genomic DNA was extracted using the TIANamp bacteria DNA kit (Spin Column) (Tiangen Biotech (Beijing) Co, Ltd., Beijing, China) following the protocol for isolating genomic DNA from bacteria. Bacterial plasmid DNA was isolated using the EZNA^®^ Plasmid DNA Mini Kit I (Omega Bio-tek Inc., Norcross, GA, USA). The concentration and purity of the DNA was measured using the Nano Drop 2000 spectrophotometer (Thermo Fisher Scientific Inc., Waltham, MA, USA). All conventional PCR reactions were carried out in a Bioer XP Thermal Cycler (Hangzhou Bioer Tech. Co., Ltd., Hangzhou, China). Amplification of the DNA was performed in 50 µL total volumes with 2× TSINGKE Master Mix (TsingKe Biological Technology, Beijing, China) while the PCR conditions were 94 °C for 10 min followed by 35 cycles of denaturation at 94 °C for 30 s, annealing at 57~62 °C (specific for each gene) for 30 s, and extension at 72 °C for 1 min/kb.

### 4.3. In Silico Analysis of Type VI Secretion Loci

A genome wide analysis was performed in this study to reveal the veil of T6SS in the *A. avenae* subsp. *avenae* strain RS-2. The components and location of T6SS homologs in Aaa strain RS-2 were determined by BLASTN and TBLASTX searching, in which T6SS information described in [11,30] was used as bait sequences against strain RS-2 genome. In addition, to identify and compare the homology of T6SS genes across *A. avenae* subsp. *avenae* strain RS-2, *A. avenae* subsp. *avenae* strain RS-1, *A. avenae* subsp. *avenae* ATCC 19860 and *A.citrulli* AAC00-1, a phylogenetic profile—which is matrix of the presence/absence of genes across the above bacteria—was created. A phylogenetic tree was built using the neighbor-joining method in MEGA6 [43] with 1000 bootstraps.

### 4.4. Generation of T6SS Mutants and Complementation

To examine the role of T6SS in the virulence mechanism of the Aaa strain RS-2, an insertional mutagenesis on target gene was generated by suicide plasmid pJP5603 [44] through homologous recombination on the background of wild-type strain RS-2. In-frame deletion of T6SS genes and their complementation were performed following the procedure of Liu et al. [16]. In brief, the internal DNA fragment of each gene of T6SS was PCR amplified using genomic DNA of wild-type strain RS-2 as template. The PCR product was cloned into pGEM-T Easy vector (Promega Corporation, Madison, WI, USA), verified by sequencing and digested with appropriate restriction enzymes (Supplementary Tables S2 and S3), and then ligated into the suicide vector pJP5603. The resulting plasmid constructs were moved into the wild-type strain RS-2 by biparental mating using *E. coli* strain S17-1 [45] as a donor. Mutant checking of 25 T6SS genes were confirmed by PCR amplification using primers flanking the genes of interest. For complementation, the whole open reading frame of these genes along with 500 bp upstream of the start codon including its native promoter were amplified by PCR using wild-type strain RS-2 as template and sub-cloned into the expression vector pRADK [46] after sequencing verification. The resulting constructs were introduced into the corresponding mutants by filter mating [47] and the complementation of the corresponding mutants was selected by Chl + Km + Rif resistance. Primers and the restriction enzymes used in making of T6SS mutants and their complementation were listed in Supplementary Table S3.

### 4.5. Seedling Pathogenicity in Rice

To determine the effect of T6SS on the seedling pathogenicity of bacterial brown stripe of rice, seed transmission assays were carried out as described in Li et al. [9] with some modifications. Briefly, germinated rice seeds (cv. II You 023, *n* = 100/mutant) were inoculated with gentle agitation using immersion in 10 mL of a cell suspension containing approximately ~1 × 10^8^ CFU/mL (OD_600_ = 0.6) of Aaa wild-type strain RS-2 and each T6SS mutant for 4 h. Seeds treated with double-distilled water (ddH_2_O) in the same manner were also used as negative control. After inoculation, bacterial suspensions were discarded and the seeds were then air dried at room temperature for 24 h. The inoculated dried seeds were planted in plate moisture method containing 0.5% agar (15 seeds per plate) and incubated under greenhouse conditions (28 ± 2 °C, 80% humidity) with a 14:10 h light–dark photoperiod. Seedling emergence and plant height was recorded after 5 days of sowing. This experiment was repeated three times with three replications of each treatment.

### 4.6. Bacterial Growth Assays

Bacterial growth was assessed by inoculating 5.0 µL cell suspensions (overnight broth cultures adjusted to OD600 = 0.6) into 5 mL of LB broth. Bacterial numbers were determined by measuring absorbance at 600 nm using a Thermo Multiskan EX Micro plate Photometer (Perkin Elmer Lambda 35 UV/VIS, Thermo Fisher Scientific Inc.) after incubation at 200 rpm, 30 °C, for 0.0, 1.5, 3.0, 6.0, 12.0, 24.0, and 48.0 h, respectively. Only the LB broth was used as the negative control.

### 4.7. Biofilm Formation Measurement

Biofilm formation assays were performed on T6SS mutants of the Aaa strain RS-2 in 96-well microtitre plates (Corning-Costar Corp., Corning, NY, USA) using the method of Peeters et al. [48]. Briefly, the overnight cell suspension of the Aaa wild-type strain RS-2 and mutants were re-cultured into a fresh LB broth containing appropriate antibiotics with a 1:100 dilution under shaking to mid exponential growth. Then each well was inoculated with 100 µL of approximately ~1 × 10^8^ CFU/mL (OD_600_ = 0.6) bacterial suspension and incubated at 30 °C for 48 h of adhesion without agitation while twelve wells filled with sterile ddH_2_O served as blanks. Culture media were then poured out and each well in the plates was washed three times with sterile ddH_2_O. Following air-dried for 30 min, each well was stained with 125 µL of 0.1% (*w*/*v*) crystal Violet (CV) solution for 45 min at room temperature. The unbound crystal Violet was removed and then washed with ddH_2_O. To solubilize the crystal Violet stained cells, 150 µL of 33% acetic acid was added into each well. Bacterial biofilm was quantified by measuring their optical density at 590 nm using a Thermo Multiskan EX Micro plate Photometer (Thermo Fisher Scientific Inc.). Twelve replications of each treatment were used for quantitative measurement in the three repeated experiments.

### 4.8. Motility Assays

Bacteria were cultured overnight in LB broth supplemented with appropriate antibiotic at 30 °C in a shaker, and then centrifuged down, washed and diluted to OD_600_ = 0.6 in sterile water. The media for motility assays was LB containing 0.3% agar for swimming as described by Liu et al. [49]. Five µL cell suspensions of wild-type strain RS-2 and mutants were spotted on the center of each swimming plate. After 48 h of incubation, the colony diameter was measured. This assay was repeated three times independently with three replications of each treatment.

### 4.9. Measurement of the Secreted Hcp by ELISA

Enzyme-linked immune sorbent assay (ELISA) was performed for the measurement of the secreted Hcp, which has been found to be associated with the adaptation to various effector and bacterial virulence [26,29,30]. The standard ELISA was conducted in a 96 microtiter plate as described by Slutzki et al. [50]. Briefly, one milliliter of overnight and 30 °C bacterial culture (approximately OD_600_ = 0.6) was harvested by centrifugation at 4000× *g* for 10 min and the supernatant was then filtered through 0.22 µm filter. The microtiter plates were coated with 150 µL of filtered antigen diluted 10 times with coating buffer and incubated overnight at 4 °C. The plates were blocked with 175 µL/well of blocking buffer (phosphate-buffered saline (PBS), 10 mM CaCl_2_, 1% bovine serum albumin (BSA), 0.05% Tween 20) for 1 h at 37 °C and then washed with washing buffer. For the detection of Hcp effector protein, the polyclonal antibody Hrp-conjugated Goat Anti-Rabbit IgG at a 1:5000 dilution from Shanghai Health Company (Shanghai, China) was used. After incubation at 37 °C for periods ranging from 2 to 24 h, the enzymatic reaction (colour development) was recorded and the optical density was measured using a microplate reader (Multiscan microtiter plate reader) set at 450 nm while absorbance (≥0.5) after subtraction of values for negative control samples were considered as positive.

### 4.10. Statistical Analyses

The software STATGRAPHICS Plus version 4.0 (Copyright manugistics Inc., Rockville, MD, USA) was used to perform the statistical analyses. The levels of significance (*p* < 0.05) of the main treatments and their interactions were calculated by analysis of variance after testing for normality and variance homogeneity.

## 5. Conclusions

The current study revealed the diversity of T6SS genes in the pathogenicity of the Aaa strain RS-2 to rice seedlings. Indeed, the virulence was attenuated by the mutation of 15 T6SS genes, but unaffected by the mutation of the other10 T6SS genes. Furthermore, this study found that the mutation of the 15 virulence-associated T6SS genes caused a significant reduction in Hcp secretion, while some of these resulted in a significant reduction in the growth, biofilm formation and swimming of the Aaa strain RS-2. In addition, our preliminary study found that there was a difference in the virulence of strain RS-1 and RS-2 although both of them have the similar T6SS of high homology, indicating that there may be other virulence factors involved in the pathogenicity of Aaa. However, this study clearly highlighted that T6SS played vital roles in the virulence of this rice pathogenic bacteria, which may function by affecting bacterial growth, biofilm formation, swimming ability and the secretion of Hcp effectors.

## Supplementary Materials

Supplementary materials can be found at www.mdpi.com/1422-0067/18/10/2024/s1.
