# Supplementary material for: Role of the Genes of Type VI Secretion System in Virulence of Rice Bacterial Brown Stripe Pathogen Acidovorax avenae subsp. avenae Strain RS-2
Source: Int J Mol Sci. 2017 Sep 21;18(10):2024. doi: 10.3390/ijms18102024 (PMC5666706; doi:10.3390/ijms18102024)
Supplement: Supplementary file 1 [file ijms-18-02024-s001.pdf]

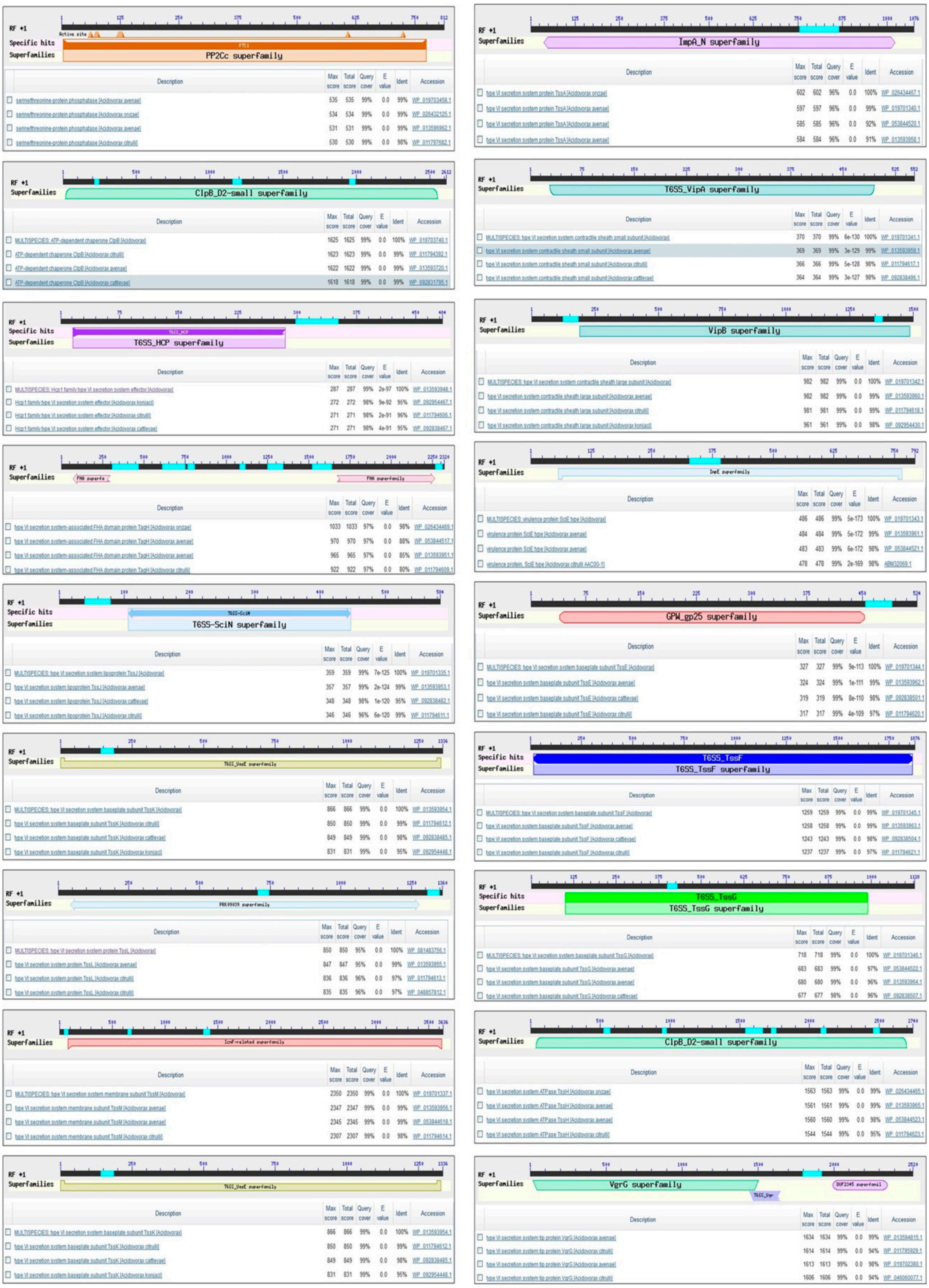

Figure S1. BLASTX images, generated in NCBI databases, showing the presence of T6SS putative proteins in *Acidovorax avenae* subsp. *avenae* strain RS-2, which were homologs in closely related bacteria such as *A. avenae* subsp. *avenae* ATCC 19860 and *Acidovorax citrulli* AAC00-1. Note: Similar results were found for vgrG 1~8 due to high sequence similarity. Here only show one verG result as an example.

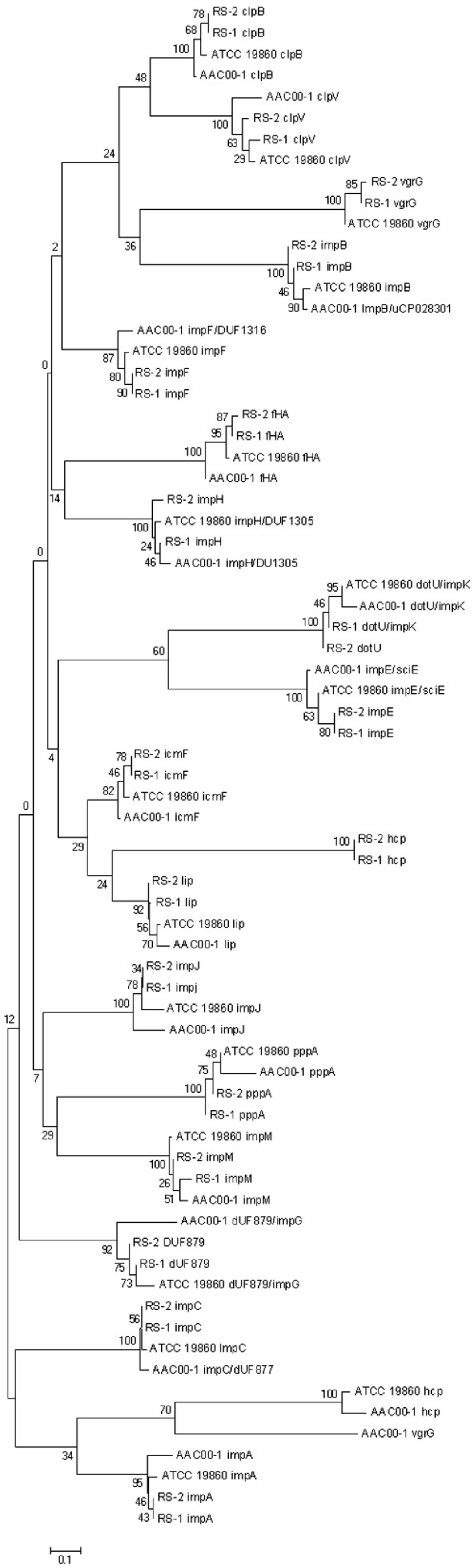

Figure S2. Phylogenetic tree, generated by the neighbour-joining method using T6SS gene sequences of bacteria from *Acidovorax avenae* subsp. *avenae* strain RS-1, RS-2, and ATCC 19860; *Acidovorax citrulli* AAC00-1, showing the homologs of T6SS among the members of the genus *Acidovorax.* The two-parameter Kimura correction of evolutionary distances was used. Bootstrap analysis (1000 replicates) for node values greater than 50% are given. Bar 0.1 substitutions per nucleotide position.

Table S1 *In silico* predictions of type VI secretion system genes in *Acidovorax avenae* subsp. *avenae* strain RS-2 genome and their sequence homologies with that of strain RS-1, and ATCC 19860 of Aaa, as well as strain AAC00-1 of *Acidovorax citrulli*.

T6SS genes	Locus Tag	Putative Functions
pppA	Acav_4620	protein serine/threonine phosphatase
clpB	Acav_1267	ATP-dependent chaperone
hcp	Acav_1504	hypothetical protein
fHA	Acav_1507	FHA domain-containing protein
lip	Acav_1509	type VI secretion lipoprotein
impJ	Acav_1510	type VI secretion protein
dotU	Acav_1511	type VI secretion protein
icmF	Acav_1512	type VI secretion protein
impM	Acav_1513	type VI secretion system-associated protein
impA	Acav_1514	type VI secretion-associated protein
impB	Acav_1515	type VI secretion protein
impC	Acav_1516	type VI secretion protein
impE	Acav_1517	type VI secretion protein
impF	Acav_1518	Lysozyme-like protein
dUF879	Acav_1519	type VI secretion protein
impH	Acav_1520	type VI secretion protein
clpV	Acav_1521	ATPase
vgrG-1	Acav_0298	type VI secretion-associated protein
vgrG-2	Acav_0662	type VI secretion-associated protein
vgrG-3	Acav_2399	type VI secretion-associated protein
vgrG-4	Acav_3111	type VI secretion-associated protein
vgrG-5	Acav_3369	type VI secretion-associated protein
vgrG-6	Acav_3676	type VI secretion-associated protein
vgrG-7	Acav_3724	type VI secretion-associated protein
vgrG-8	Acav_1905	type VI secretion-associated protein

Table S2 Strains and plasmids used in this study.

Strain or plasmid	Relevant characteristics [a]	Sources or references
	***Acidovorax avenae* subsp. *avenae* strains**	
RS-2	Rif^R; The pathogen of bacterial brown stripe of rice, isolated from the diseased rice from Zhejiang province in China	Lab collection
ΔpppA	Rif^R, Kan^R; RS-2 in-frame deletion mutation defective in *pppA*	This study
ΔpppA-comp	Rif^R, Amp^R, Kan^R; Δ*pppA* complemented with pRADK-*pppA*	This study
ΔclpB	Rif^R, Kan^R; RS-2 in-frame deletion mutation defective in *clpB*	This study
ΔclpB-comp	Rif^R, Amp^R, Kan^R; Δ*clpB*complemented with pRADK-*clpB*	This study
Δhcp	Rif^R, Kan^R; RS-2 in-frame deletion mutation defective in *hcp*	This study
Δhcp-comp	Rif^R, Amp^R, Kan^R; Δ*hcp* complemented with with pRADK-*hcp*	This study
ΔfHA	Rif^R , Kan^R; RS-2 in-frame deletion mutation defective in *fHA*	This study
Δlip	Rif^R, Kan^R; RS-2 in-frame deletion mutation defective in *lip*	This study
ΔimpJ	Rif^R, Kan^R; RS-2 in-frame deletion mutation defective in *impJ*	This study
ΔimpJ-comp	Rif^R, Amp^R, Kan^R; Δ*impJ* complemented with pRADK-*impJ*	This study
ΔdotU	Rif^R, Kan^R; RS-2 in-frame deletion mutation defective in *dotU*	This study
ΔdotU-comp	Rif^R, Amp^R, Kan^R; Δ*dotU* complemented with pRADK- *dotU*	This study
ΔicmF	Rif^R, Kan^R; RS-2 in-frame deletion mutation defective in *icmF*	This study
ΔicmF-comp	Rif^R, Amp^R, Kan^R; Δ*icmF* complemented with pRADK-*icmF*	This study
ΔimpM	Rif^R, Kan^R; RS-2 in-frame deletion mutation defective in *impM*	This study
ΔimpM-comp	Rif^R, Amp^R, Kan^R; Δ*impM* complemented with pRADK-*impM*	This study
ΔimpA	Rif^R, Kan^R; RS-2 in-frame deletion mutation defective in *impA*	This study
ΔimpB	Rif^R, Kan^R; RS-2 in-frame deletion mutation defective in *impB*	This study
ΔimpC	Rif^R, Kan^R; RS-2 in-frame deletion mutation	This study

	defective in *impC*	
ΔimpE	Rif^{R}, Kan^{R}; RS-2 in-frame deletion mutation defective in *impE*	This study
ΔimpF	Rif^{R}, Kan^{R}; RS-2 in-frame deletion mutation defective in *impF*	This study
ΔdUF879	Rif^{R}, Kan^{R}; RS-2 in-frame deletion mutation defective in *dUF879*	This study
ΔimpH	Rif^{R}, Kan^{R}; RS-2 in-frame deletion mutation defective in *impH*	This study
ΔclpV	Rif^{R}, Kan^{R}; RS-2 in-frame deletion mutation defective in *clpV*	This study
ΔvgrG-1	Rif^{R}, Kan^{R}; RS-2 in-frame deletion mutation defective in *ΔvgrG-1*	This study
ΔvgrG-2	Rif^{R}, Kan^{R}; RS-2 in-frame deletion mutation defective in *ΔvgrG-2*	This study
ΔvgrG-3	Rif^{R}, Kan^{R}; RS-2 in-frame deletion mutation defective in *ΔvgrG-3*	This study
ΔvgrG-4	Rif^{R}, Kan^{R}; RS-2 in-frame deletion mutation defective in *ΔvgrG-4*	This study
ΔvgrG-5	Rif^{R}, Kan^{R}; RS-2 in-frame deletion mutation defective in *ΔvgrG-5*	This study
ΔvgrG-6	Rif^{R}, Kan^{R}; RS-2 in-frame deletion mutation defective in *ΔvgrG-6*	This study
ΔvgrG-7	Rif^{R}, Kan^{R}; RS-2 in-frame deletion mutation defective in *ΔvgrG-7*	This study
ΔvgrG-8	Rif^{R}, Kan^{R}; RS-2 in-frame deletion mutation defective in *ΔvgrG-8*	This study
	***Escherichia coli* strains**	
DH5*α*	F-Φ80d *lac*ZΔM15Δ(*lac*ZYA-*arg*F) U169 *recA1endA1*, *hsdR17*(rk−, mk+) *phoAsupE44 λ− thi*-1 *gyrA96rel*A1	Invitrogen
S17-1 *λ pir*	*λ* Lysogenic S17-1 derivative producing π protein for replication of plasmids carrying *oriR6K*; *recAprohsdR*RP4-2-Tc::Mu-Km::Tn7 *λ-pir*	Simon et al., 1983
	**Plasmids**	
pJP5603	Kan^{R};R6K-based suicide vector; requires the *pir*-encoded π protein for replication	Penfold and Pemberton, 1992
pGEM-T	Amp^{R}; cloning vector	Promega
pRADK	Amp^{R}，Chl^{R}，Km^{R}; broad host expression vector	Gao et al., 2005

[a]Amp^{R}, Kan^{R}, Rif^{R}, Chl^{R} indicate resistant to Ampicillin-, Kanamycin-, Rifampicin-, Chloramphenicol -, respectively.

Table S3 List of primers used in this study.

Primers	Nucleotide sequences (5′-3′)	Characterization
pppA-F	CGCGGATCCAAGCCTGCGTGGACT (B)	324 bp internal upstream fragment of *pppA*; used to create *ΔpppA*
pppA-R	GGAATTCGCACCTGGAACTCATTG (E)	
clpB-F	CGGGATCCGGACGAGGGACAGAC(B)	339 bp internal upstream fragment of *clpB*; used to create *ΔclpB*
clpB-R	CGGAATTCATGTAGCGGTGGGAC(E)	
hcp-F	CGGGATCCGACCGACATCCGTTCC(B)	235 bp internal upstream fragment of *hcp*; used to create *Δhcp*
hcp-R	GGAATTCGGTGAGGGTGATCTTGC(E)	
fHA-F	CGGGATCCTCAGCCCTGTTTCCA(B)	237 bp internal upstream fragment of *fHA*; used to create *ΔfHA*
fHA-R	CGGAATTCTTTCCTCGCTCTTGC(E)	
lip-F	CGGGATCCCAGCAACCTGAACCG(B)	192 bp internal upstream fragment of *lip*; used to create *Δlip*
lip-R	CGGAATTCGCCTCGTCCTTGTCC(E)	
impJ-F	CGGGATCCAACAGGACCGCTACACC(B)	432 bp internal upstream fragment of *impJ*; used to create *ΔimpJ*
impJ-R	CCGGAATTCGGCTCACCCACCCAT(E)	
dotU-F	CGGGATCCGGCATTCCAGCATTAC(B)	303 bp internal upstream fragment of *dotU*; used to create *ΔdotU*
dotU-R	CGGAATTCAAACCGAGCGTCAGG(E)	
icmF-F	CGGGATCCCGGCAACAACCAGAA(B)	766 bp internal upstream fragment of *icmF*; used to create *ΔicmF*
icmF-R	GGAATTCCTCATGCGAGAAAACG(E)	
impM-F	CGGGATCCCGCTGCTATCCGCTCAC(B)	278 bp internal upstream fragment of *impM*; used to create *ΔimpM*
impM-R	CCGGAATTCAACATCGCCTCGCCAC(E)	
impA-F	CGGGATCCAGGACGGCGACTACTT(B)	355 bp internal upstream fragment of *impA*; used to create *ΔimpA*
impA-R	CCGGAATTCTCGTTCAGCACCACC(E)	
impB-F	CGGGATCCTGATGGCGGACCTCT(B)	147 bp internal upstream fragment of *impB*; used to create *ΔimpB*
impB-R	CGGAATTCCCGTCAGCGTGTTGG(E)	
impC-F	CGGGATCCGGACCAGAGCCCTAT(B)	468 bp internal upstream fragment of *impC*; used to create *ΔimpC*
impC-R	CGGAATTCAACGAGCGGTTGATG(E)	
impE-F	CGGGATCCCTCGGATGGATGGTG(B)	292 bp internal upstream fragment of *impE*; used to create *ΔimpE*
impE-R	CGGAATTCCGTTCTGGAGCGTGA(E)	
impF-F	CGCGGATCCGCCGAAGCGGTCTAT(B)	307 bp internal upstream

impF-R	CGGAATTCGCAGGGTGTTGTGGTG(E)	fragment of *impF*; used to create *ΔimpF*
dUF879-F	CGCGGATCCTTTCGTGCCGTTCTT(B)	396 bp internal upstream fragment of *dUF879*; used to create *ΔdUF879*
dUF879-R	GGAATTCGCGAGGTAGTTGAGGG(E)	
impH-F	CGCGGATCCATCCGCATCCGTTCG(B)	226 bp internal upstream fragment of *impH*; used to create *ΔimpH*
impH-R	GGAATTCCCGTTGGGACCATAGAG(E)	
clpV-F	CGGGATCCGCTATGTGGGCTACG(B)	526 bp internal upstream fragment of *clpV*; used to create *ΔclpV*
clpV-R	CGGAATTCCATCGACCGAACTCT(E)	
vgrG-1-F	CGGGATCCCCGACCGTGAGAATA(B)	564 bp internal upstream fragment of *vgrG-1*; used to create *ΔvgrG-1*
vgrG-1-R	CGGAATTCGGGGTTTTGGAGTTT(E)	
vgrG-2-F	CGCGGATCCTCGTGGAAGAGGTTT(B)	367 bp internal upstream fragment of *vgrG-2*; used to create *ΔvgrG-2*
vgrG-2-R	CGGAATTCGCTTGGCACTGTTGAA(E)	
vgrG-3-F	CGGGATCCACCAGAAGGGGATAGAG(B)	424 bp internal upstream fragment of *vgrG-3*; used to create *ΔvgrG-3*
vgrG-3-R	CGGAATTCTTGGGACTGATGAGGCT(E)	
vgrG-4-F	CGGGATCCTCGTGGAAGAGGTTTTGA(B)	574 bp internal upstream fragment of *vgrG-4*; used to create *ΔvgrG-4*
vgrG-4-R	CGGAATTCGATGAGGTACTGCTGGTT(E)	
vgrG-5-F	CGGGATCCCATCGCCTCTACTCCTAC(B)	637 bp internal upstream fragment of *vgrG-5*; used to create *ΔvgrG-5*
vgrG-5-R	CGGAATTCCTGCTCTTCGTTGTGCTT(E)	
vgrG-6-F	CGCGGATCCCGCCTGGGATTACTG(B)	593 bp internal upstream fragment of *vgrG-6*; used to create *ΔvgrG-6*
vgrG-6-R	CGGAATTCGCTGTTGAACGAGGAA(E)	
vgrG-7-F	CGGGATCCCGCCGAACTGAACAA(B)	737 bp internal upstream fragment of *vgrG-7*; used to create *ΔvgrG-7*
vgrG-7-R	CGGAATTCGCAGCCCAGGATGAT(E)	
vgrG-8-F	CGGGATCCCAACGACGACTACGAC(B)	367 bp internal upstream fragment of *vgrG-8*; used to create *ΔvgrG-8*
vgrG-8-R	CCGGAATTCAGAGCCCTCTTCCTG(E)	
Aaa-F<br>Aaa-R	GACCAGCCACACTGG GAC<br>CTGCCGTACTCCAGCGAT	370 bp *A. oryzae* RS-2 special primers used to screen *A. oryzae*
pJP5603-F<br>pJP5603-R	CTGATGCCGCCGTGTTC<br>CCAATAGCAGCCAGTCCCT	112 bp specific primers of pJP5603 for confirming the mutant
16s rDNA-F<br>16s rDNA-R	AGAGTT TGATCCTGGCTCAG<br>GGTTACCTTGTTACGACT T	1000 bp specific primers of bacterial for checking

		the strain sequence
pppA-comp-F	TGCCATGGTACCCGGGAGCTCGGATGGCGGCATTGAGCG (S)	1310 bp fragment using for complementation of *pppA* mutant
pppA-comp-R	CGCGTCTGCATGTGGAAGCTTTCAATCGGTACGTAGCAACAATCG(H)	
clpB-comp-F	TGCCATGGTACCCGGGAGCTCCCGTAACAGAACCCGACAGC(S)	2110 bp fragment using for complementation of *clpB* mutant
clpB-comp-R	CGCGTCTGCATGTGGAAGCTTTTACCCCACAGCCGCCGC(H)	
hcp-comp-F	TGCCATGGTACCCGGGAGCTCCCCTTGGAACCCAGGTAGGG(S)	983 bp fragment using for complementation of *hcp* mutant
hcp-comp-R	CGCGTCTGCATGTGGAAGCTTTTACATTTCCTTGTTGCCCTTGA(H)	
dotU-comp-F	TGCCATGGTACCCGGGAGCTCCGGCCCGCGGCCATGCCG(S)	1853 bp fragment using for complementation of *dotU* mutant
dotU-comp-R	CGCGTCTGCATGTGGAAGCTTTTAGTTCTTCGGTGTGCCGG(H)	
impJ-comp-F	TGCCATGGTACCCGGGAGCTCGTGCCGCCGGCCGTCTTC(S)	1835 bp fragment using for complementation of *impJ* mutant
impJ-comp-R	CGCGTCTGCATGTGGAAGCTTTCAGCGCCGTATGGCCCA(H)	
icmF-comp-F	TGCCATGGTACCCGGGAGCTCGCCAGTTCCTGGAGCCCG(S)	4133 bp fragment using for complementation of *icmF* mutant
icmF-comp-R	CGCGTCTGCATGTGGAAGCTTTCAGAGATTGCCAGGACACGC(H)	
impM-comp-F	TGCCATGGTACCCGGGAGCTCCGCTTCCGCCAGGGTGTC(S)	1196 bp fragment using for complementation of *impM* mutant
impM-comp-R	CGCGTCTGCATGTGGAAGCTTCTATGCATTCGGGCCTCCG(H)	

[a] Underlined nucleotides in some of the PCR primers represent restriction sites of enzymes indicated in parentheses (B = *Bam*HI; E = *Eco*RI; S = *Sac*I; H: *Hind*III).

Table S4 Effect of type VI secretion system on the plant height of *Acidovorax avenae* subsp. *avenae* strain RS-2 to rice seedling.

Strains	Plant height (cm)	Decrease (%)	Strains	Plant height (cm)	Decrease (%)
ddH_2O	5.78 ± 0.10**	--	*ΔvgrG-7*	3.25 ± 0.21*	43.77
ΔpppA	4.01 ± 0.16**	30.62	*ΔvgrG-8*	3.57 ± 0.05*	38.24
ΔclpB	4.41 ± 0.17**	23.70	*ΔfHA*	2.95 ± 0.07	48.96
Δhcp	5.41 ± 0.16**	6.40	*ΔdUF879*	2.93 ± 0.32	49.31
ΔimpJ	4.46 ± 0.08**	22.84	*ΔimpC*	3.07 ± 0.79	46.89
ΔdotU	4.09 ± 1.06**	29.24	*ΔimpA*	2.58 ± 0.27	55.36
ΔicmF	4.83 ± 0.20**	16.44	*ΔclpV*	2.95 ± 0.22	48.96
ΔimpM	4.76 ± 0.22**	17.65	*ΔimpF*	2.67 ± 0.23	53.81
ΔvgrG-1	3.49 ± 0.26*	39.62	*ΔimpB*	2.92 ± 0.08	49.48
ΔvgrG-2	3.44 ± 0.14*	40.48	*ΔimpE*	2.41 ± 0.12	58.30
ΔvgrG-3	3.57 ± 0.15*	38.24	*Δlip*	2.86 ± 0.10	50.52
ΔvgrG-4	3.16 ± 0.05*	45.33	*ΔimpH*	3.04 ± 0.12	47.40
ΔvgrG-5	3.57 ± 0.05*	38.24	wild-type	2.15 ± 0.08	62.80
ΔvgrG-6	3.53 ± 0.15*	38.93			

"*" $0.01 < p < 0.05$, significant difference; "**" $p < 0.01$, very significant difference; not marked with $0.05 < p$, no significant difference. The wild-type and ddH_2O were used as the positive and negative control, respectively.
